# Determinants of sexually transmitted infections among adolescent girls and young women in artisanal and small-scale mining communities of Uganda

**DOI:** 10.1186/s12889-025-23159-4

**Published:** 2025-05-24

**Authors:** Betty Kwagala, Hanna Chidwick, Deborah Mensah, John Bosco Asiimwe, Stephen Ojiambo Wandera, Miriam Mutabazi, Fred Ngabirano, Lydia Osei, Lydia Kapiriri

**Affiliations:** 1 Department of Population Studies, Makerere University, Kampala, Uganda; 2 Global Health, Faculty of Health Sciences, McMaster University, Hamilton, Canada; 3 Northern Empowerment Association/GRID, Tamale, Ghana; 4 Uganda Christian University, Mukono, Uganda; 5 Ministry of Gender, Labour and Social Development, Kampala, Uganda; 6 Department of Geography, University of Ghana, Accra, Ghana; 7 Department of Health, Aging & Society, McMaster University, Hamilton, Canada

**Keywords:** Young female artisanal mine workers, Sexually transmitted infections, Uganda

## Abstract

**Background:**

The artisanal and small-scale mining (ASM) sector has become an important employer in mineral rich countries of sub Saharan Africa where women constitute up to half of the labour force. However, gender and socio-economic marginalization negatively impact the sexual and reproductive health (SRH) of the adolescent girls and young women (AGYW) who work in the ASM sector. Despite the growing literature on adolescents’ SRH, there is a paucity of literature on the SRH of this last mile population. This paper fills this gap in the literature by examining the prevalence and determinants of self reported sexually transmitted infection (STI) status among AGYW in the ASM gold mining sectors of Uganda.

**Methods:**

The paper is based on 636 AGYW working in the mining sectors in Uganda who had ever had sex. Descriptive analysis involved frequency distributions and chi squared tests. Multivariable analysis involved fitting a binary logistic regression model to assess the determinants of self reported STI status of the AGYW.

**Results:**

Almost half (47%) of the respondents had a self reported STI during the 12 months preceding the study. The odds of reporting an STI were higher among adult young women compared with minors (AOR = 3.35; 95% CI 1.82 – 6.16); AGYW with primary level of education compared to those with none (AOR = 2.89; 95% CI 1.24 – 6.75); who drank alcohol (AOR 1.59; 95% CI 1.06—2.39); and engaged in transactional sex (AOR 2.42; 95% CI 1.37 – 4.28).

**Conclusions:**

The results highlight the urgent need to respond to the high prevalence of self reported STIs among AGYW in ASM. The risk factors constitute multiple and intersecting vulnerabilities that require both preventive and curative interventions targeting female and male ASM workers and host communities, with emphasis on behavioral change and promotion of viable alternative sources of income. The ministries of Health, Gender, Labour and Social Development and key development partners should adopt a multi sectoral approach that effectively engages key stakeholders, including mining host communities, given the close interrelations between gender, health and economic aspects of the AGYW’s lives.

## Background

Artisanal and small-scale mining (ASM) is an important source of minerals globally [1, 2]. The sector is a significant employer in low and middle income countries in particular. In many sub-Saharan African countries, ASM provides employment to many including vulnerable groups such as women and adolescents [3, 4]. Uganda is endowed with a variety of minerals, including gold, which is mainly extracted through ASM. Women constitute about 50% of the workforce [2, 3, 5–7]. The involvement of poor adolescent girls and young women (AGYW) in a male dominated informal sector raises concerns over their economic well-being and sexual and reproductive health (SRH), including the risk of contracting Sexually Transmitted Infections (STIs) [8, 9].

STIs are among the most important global reproductive health challenges given their significant (reproductive) health and socio-economic impacts. More than one million infections occur globally on a daily basis [10]. The STI prevalence among women of reproductive age for Uganda is 24% [11]. SRH studies in sub-Saharan Africa report a high prevalence of STIs in general, and in mining communities in particular [9, 12–17]. AGYW in the mining sector are particularly at risk given their experience of multiple, intersecting vulnerabilities associated with a young age, gender and economic status [1–3, 12, 17–23]. Contextual gender norms and the hierarchical gender relations that accord women a subordinate role in relationships are closely linked to proximate factors that are associated with STI status of women [10].

STIs in mining communities in sub-Saharan Africa tend to be concentrated amongst the youth, making age and important determinant [13]. Studies show that AGYW in mining communities are more vulnerable to sexual exploitation [2, 3, 5, 17]. The high prevalence of STIs is also attributed to challenges of accessing SRH services and information due to stigma and sociocultural barriers [9]. In addition, religion plays an important role in influencing sexual behavior [24–26]. Findings of a Ugandan study among married women of reproductive age revealed that Muslim women had higher odds of reporting an STI compared with Catholics women [26].

Contracting an STI is also influenced by behavioral and other factors such as migration, alcohol consumption, access to SRH information, economic status and experience of sexual violence. Mine work attracts national and international migrant workers [13, 27]. Studies show an association between migration status and STIs. Migrant workers usually have minimal social support [17] as their work involves prolonged stays away and isolation from their families and consistent sexual partners, thus encouraging new sexual partnerships which potentially increase the risk of STIs [9, 12, 14, 16]. Alcohol and substance misuse in mining communities contribute to risky sexual behaviour and thus STIs, since their consumption often involves unprotected sex [9, 12]. In addition, AGYW in the ASM sector, have limited information on SRH, and limited safer sex negotiating power, which increases their vulnerability to STIs [9, 12, 28]. Similarly, an individual’s economic status can impact sexual behavior as poverty and economic dependence, limit women’s safer sex negotiation power. Poverty in poor mining communities induces transactional sex, a key risk factor for STIs [17]. Limited avenues for entertainment in mining communities combined with low incomes contribute to transactional sex and sex with multiple partners, which can result in STIs [9, 13, 15, 17, 28–30]. Sex and gender based violence that is often reported in mine settings, also exposes women to STIs [9, 31, 32]. Sexual violence is rarely protected, and has immediate and far reaching impacts [33–35]. Gender based violence disempowers women and often results in risky sexual behaviors such as multiple sexual partnerships, transactional sex and early marriage which increase the risk for STIs [33].

Studies on ASM in Uganda have not addressed the unique SRH experiences of the AGYW in the sector using quantitative data. This paper fills an important gap in literature and contributes to fulfilment of Sustainable Development Goal three which highlights the need to leave no one behind in the development process [36]. The aim of this paper is to examine the prevalence and factors associated with STI status among AGYW that work in the ASM.

## Methods

### Study design

This paper is based on findings of a cross sectional household survey that was conducted among AGYW in the ASM sector in Uganda.

### Study sites

The study was conducted in ASM communities of Busia and Namayingo districts, Eastern region, and Kassanda and Mubende districts, central region. The choice of specific sites was based on the existence of gold ASM activities, and feasibility of implementing the study, including security.

### Sample size and method of data collection

We focused on AGYW age 10–24 years, living in the ASM communities. We interviewed 810 respondents. The sample size were determined using Yamane’s [37] formula, taking into consideration the standard unknown design effect of 2. Previous national surveys have shown a response rate of 98%, which was the basis of the anticipated response rate (98%.). The analysis is based on the 636 AGYW who had ever had sex.

Eligible respondents were AGYW who either directly worked or provided services to ASM workers. Service providers were included because they derived their livelihoods from the mines, are affected by their close interactions with ASM workers and often end up working in the mines. Adolescents as young as 10 years were considered because adolescents in the ASM sector are at a high risk of defilement. In Uganda, 12% of adolescents in the general population had their sexual debut below the age of 15 years [11]. It is anticipated that the percentage is higher among adolescents that engage in ASM [31].

In the selected locations, active gold mining sites were included in the study. Since the target age group engages in activities that are rarely licensed and are migratory [38], all households with eligible AGYW at the time of data collection were taken into consideration. There was no gold rush when data were collected, which posed some challenges in identifying eligible respondents. Due to limited number of ASM in the locations, and the illegal nature and stigmatization of the activities, we recruited respondents from each sub region until a required sample was achieved. We interviewed 810 AGYW in Uganda (405 in each region) [11] and achieved the targeted sample with at least 98% response rate.

#### Data collection

Research teams were trained and the study tool was pretested prior to data collection. We used the KoBo Collect tool that functions even in remote communities with limited internet connectivity, to collect data. Survey data were collected between March and May 2023. Questions addressing STI status and key background factors were adapted from the Uganda Demographic and Health Survey women’s validated questionnaire [39].

Interviews were conducted at household level. One AGYW was interviewed per household. If a household had more than one eligible AGYW, simple random selection was applied to select the respondent. We used the lottery method where names were listed on paper and random selection done using the fish bowl method. Data were collected by young female research assistants to match the prospective respondents. In order to ensure privacy, interviews were conducted in a private but open place where any approaching persons could be observed. The interviews were conducted in the relevant local languages.

#### Variables and measurements

The dependent factor self reported STI status was coded as a binary outcome. Respondents were asked whether during the year preceding the study they had a) a bad smelling/abnormal genital discharge, b) a genital sore or ulcer or c) a disease acquired through sexual contact [39]. “Yes” responses to one or more of the questions were coded as 1 and “No” responses to all three questions were coded as 0.

Independent factors included: age, religion, marital status, migration status, education level, kind of work in the mine, employer, fairness of payment, whether the respondent had savings, membership of a saving/investment group, number of sexual partners, alcohol consumption, transactional sex, and experience of physical violence, sexual violence in the past one year. Generation of the variables—physical and sexual violence was based on the following questions tailored after the Demographic and Health Survey. Several questions were asked to constitute physical and sexual violence. Questions to address physical violence included the following- Did anybody: Push you? Shake you, or throw something at you? Slap you? Twist your arm or pull your hair? Punch you with his fist or with something that could hurt you? Kick you, drag you, or beat you up? Try to choke you or burn you on purpose? and Threaten or attack you with a knife, gun, or other weapon? For sexual violence, respondents were asked: Did anybody: Physically force you to have sex with him when you did not want to? Physically force you to perform any other sexual acts you did not want to? Force you with threats or in any other way to perform sexual acts you did not want? If a respondent answered ‘yes’ to any of the questions, then it constituted that particular type of violence [11].

#### Data analysis

Data were analyzed using Stata statistical software version 15. Analyses involved frequency distributions to describe the characteristics of the respondents, bivariate (Chi-squared tests) to identify variables for inclusion in the multivariable analysis, followed by logistic regression analysis to isolate significant determinants of AGYW’s STI status. The results are reported at 95% confidence intervals (the level of significance was set at *p* < 0.05) (18).

#### Ethical considerations

The study was approved by the research ethics committees in Uganda (The AIDS Support Organization-2022–169 and registered by the Uganda National Council for Science and Technology- UNCST – SS149ES). Ethical clearance was also obtained from collaborating institutions – namely, McMaster University, Canada and University of Ghana. Voluntary informed consent was obtained from the adult respondents and assent from minors, after seeking consent of their caregivers where applicable. Working minors that lived alone were treated as emancipated minors. Owing to the recent COVID 19 and Ebola epidemics in Uganda, in adherence the Standard Operating Procedures to limit transmission through physical contact, we used verbal consent. Participants were assured of confidentiality**.**

## Results

### Respondents background characteristics

We interviewed 810 respondents in the two countries of which 636 (79%) had ever had sex. The analysis was based on the 1,338 respondents who had ever had sex.

Results in Table 1 show that close to half (47%) of the respondents reported that they had an STI or STI symptoms. About 89% were adults age 18–24 years. Muslims constituted close to half of the respondents (46%). The majority were migrants (60%), currently of previously married (53%), had primary or no formal education (61%), were employed by private individuals (52%), and worked in the service sector most of the time (73%). Over one in four (28%) had more than one sexual partner during the two years preceding the survey, and 23% drank alcohol. In terms of violence, 21% reported experiencing physical violence, 12% sexual violence, and 11% participated in transactional sex during the 12 months preceding the study.

Results in Table 2 show significant variations between self reported STI status and age, migration status, marital status, level of education, number of sexual partners, alcohol consumption and transactional sex. Higher proportions of self reported STIs were evident among AGYW age 18 years and above (49%), migrants (50%), previously married (61%), primary school level of education (51%), AGYW with more than one sexual partner (55%), who consumed alcohol (61%), and engaged in transactional sex (68%). Self reported STI status did not significantly vary by religion, type of employer, work done in the mines, whether the respondent worked during the COVID19 lockdown, experience physical or sexual violence.

**Table 1:** Percentage distribution of respondents by background characteristics (*N* = 636)

**Variables**	**Frequency (n)**	**Percent (%)**
**STI status**		
No STI	339	53.3
Had an STI	297	46.7
**Age**		
< 18 years	69	10.9
18 + years	567	89.1
**Religion**		
Catholic	199	31.3
Other Christians	116	18.2
Muslim	299	47.0
Others	22	3.5
**Migration status**		
Migrated	382	60.1
Not Migrated	254	39.9
**Current marital status**		
Single never married	299	47.3
Married	254	39.9
Previously married	81	12.7
**Level of education**		
None	35	5.5
Primary	354	55.7
Secondary	247	38.8
**Type of employer**		
Self-employed	282	44.3
Employed-private persons	332	52.2
Parents and Others family members	22	3.5
**Work done in the mines**		
Directly works in processing most of the time	171	26.9
Services most of the time	465	73.1
**Worked During COVID**		
No	377	59.3
Yes	259	40.7
**Number of sexual partners**		
One or none	460	72.3
Than one sexual	176	27.7
**Alcohol**		
No	490	77.0
Yes	146	23.0
**Physical violence**		
Not experienced	503	79.1
Experienced violence	133	20.9
**Sexual violence**		
Not experienced	557	87.6
Experienced violence	79	12.4
**Transactional sex**		
No	565	88.8
Yes	71	11.2
**Total**	**636**	**100**

**Table 2:** Self-reported STI status by respondents’ background characteristics (N =636

**Independent Factors**	**STI status – Had an STI?**		**P value**
	**No **	**Yes **	
**Age**			P=0.000
<18 years	75.4	24.6	
18+ years	50.6	49.4	
**Religion**			P = 0.514
Catholic	50.0	50.0	
Other Christians	56.9	43.1	
Moslem	53.2	46.8	
Others	63.6	36.4	
**Migration status**			P = 0.027
Migrated	49.7	50.3	
Not Migrated	58.7	41.3	
**Current marital status**			P = 0.019
Single never marri	53.5	46.5	
Married	57.5	42.5	
Previously married	39.5	60.5	
**Level of education**			P = 0.002
None	77.1	22.9	
Primary	48.6	51.4	
Secondary	56.7	43.3	
**Type of employer **			P = 0.595
Self-employed	55.0	45.0	
Employed-private person	51.5	48.5	
Parents and Others fa	59.1	40.9	
**Work done in mining**			P = 0.112
Directly works in	58.5	41.5	
Services & others	51.4	48.6	
**Worked during the lockdown**	100		P = 0.112
No	51.7	48.3	
Yes	55.6	44.4	
**Number of sexual partners in the past year **			P = 0.014
None or one	56.3	43.7	
Two or more partners	45.4	54.6	
**Drinks alcohol**			P=0.000
No	57.5	42.5	
Yes	39.0	61.0	
**Physical violence**			P=0.250
No	54.5	45.5	
Yes	48.9	51.1	
**Sexual violence**			P = 0.454
Not experienced	53.9	46.1	
Experienced violence	49.4	50.6	
**Transactional sex**			P = 0.000
No	55.9	44.1	
Yes	32.4	67.6	
**Total**	**53.3**	**46.7**	

### Determinants of STI status

Multivariable analysis involved assessing the determinants of STI status through fitting a logistic regression model. All variables with a p-value less than 0.2 were included in the models. We used Pearson’s correlation test to rule out multicollinearity.

Self reported STI status was significantly associated with age, level of education, alcohol consumption, and transactional sex (see Table 3). The odds of reporting an STI were higher among AGYW 18 years and above compared to those below 18 years (AOR = 3.35; 95% CI 1.82—6.16); AGYW with primary level of education compared to those with no formal education (AOR = 2.89; 95% CI 1.24—6.75), those who consumed alcohol compared to those that did not (AOR 1.59; 95% CI 1.06—2.39), and those who engaged in transactional sex compared to those that did not (AOR 2.42; 95% CI 1.37—4.28). Migration status, type of work, whether the respondent worked during the COVID19 lockdown and number of sexual partners were not significantly associated with self reported STI status.

## Discussion

Nearly half of AGYW working in the ASM sector who had ever had sex (47%) had a self-reported STI or symptoms of STIs. This is much higher than the national prevalence for Uganda (24%) [39]. This high STI prevalence amongst this last mile population requires urgent attention since the AGYW in mining communities exhibit high levels of vulnerability with respect to poverty, limited rights awareness, limited knowledge on STIs, and access to the requisite health services [21]. This result aligns with findings of previous studies that focused on mining communities in sub Saharan Africa [9, 12–14, 16].

Age was significantly associated with reporting self-reported STIs with higher odds among young women (18 years or above). Despite relatively high prevalence of early sex (33%), it is apparent that adults who have attained the age of consent exhibit less restraint in engaging in (risky) sexual activities. Our results differ from previous studies such as Ginindza et al. [40] where the odds of reporting STIs reduced with increase in age in Swaziland and Nankinga et al. [41] where age was not a significant risk factor for STIs among women of reproductive age in Uganda. The level of education of an AGYW was a significant risk factor for self reported STIs for Uganda. The higher odds of STIs among AGYW with primary education compared to those with no formal education implies inconsistency in the influence of level of education on STI status. This finding aligns with existing literature. A previous study among women in Uganda found no significant relationship between education and self-reported STIs [26]. Our findings also align with a systematic review addressing causal effects of education on SRH in low and middle-income countries which found minimal effects of and inconsistent linkages between education on SRH [42].

Results show that AGYW who consumed alcohol had increased odds of reporting an STI. Globally, Uganda is among the countries with high levels of alcohol consumption [43]. In the current study, alcohol consumption in Uganda stands at 23% (see Table 3). Existing knowledge aligns with our findings noting that alcohol consumption compromises the capacity for rational decision making and physical self-defense which could lead to risky sexual behaviors, and is a precursor to sexual violence, unprotected sex and thus STIs [44–48].

**Table 3:** Adjusted Odds Ratios (AORs) for self reported STIs among AGYW in the ASM sector in Uganda

**Variables**	**AORs**	**95% CI**
**Age** (RC = Below 18 years)		
18 years and above	3.35***	[1.82—6.16]
**Migration status** (RC = Migrant)		
Non migrant	0.81	[0.57—1.15]
**Level of education** (RC = None)		
Primary	2.89*	[1.24—6.75]
Secondary and above	1.94	[0.83—4.54]
**Work type** (RC = Works in processing)		
Services & others	1.32	[0.89—1.96]
**Worked during the COVID 19 lockdown** (RC = No)		
Yes	0.93	[0.65—1.33]
**Number of sexual partners** (RC = None or one)		
Two or more	1.34	[0.91—1.96]
**Alcohol consumption** (RC = No)		
Yes	1.59*	[1.06—2.39]
**Transactional sex** (RC = No)		
Yes	2.42**	[1.37—4.28]
Number of observations	636	

*AOR* Adjusted Odds Ratio, *CI* Confidence Interval, *RC* Reference Category

^***^p < 0.001, **p < 0.01, *p < 0.05

According to our findings, transactional sex increased the odds of self-reported STIs among Ugandan AGYW. Transactional sex is practiced in contexts where sustained negotiation for safer sex is likely to be limited, especially when it involves poor or economically dependent AGYW [13, 17]. The high prevalence of poverty among mining host communities compels AGYW to engage in transactional sex, which exposes them to STIs. Existing literature also highlights the marginalization of AGYW on the basis of gender, age, poverty and the fact that they work in a context with limited protection and guidance, which exposes them to sexual exploitation and STIs [1–3, 12, 22].

### Limitations of the study

This study has some limitations. STI status was self-reported and not verified since laboratory tests were beyond the scope of the study. Hence, there is a possibility of underreporting owing to social desirability and or poor understanding of the symptoms. This was mitigated by (i) first asking general questions that do not directly relate to STI; and (ii) describing the STI symptoms when asking the questions. There are limitations concerning randomization, since all eligible AGYW all were interviewed; the AGYW in ASM could not be readily identified in the general population because the activity is stigmatized. This erodes the generalizability of findings. However, interviewing all eligible AGYW contributed to comprehensiveness and accuracy of the findings. Since this was a cross sectional study there were no provisions for determining causal relationships. Nevertheless, findings of this paper fill a gap in highlighting the prevalence of STIs and associated factors for a neglected population in Uganda. Finally, obtaining verbal consent was a standard operating procedural requirement owing to an aftermath of epidemics. Although it was a requirement by the ethics review committees the process presents an ethical limitation.

## Conclusions

This study highlights the urgent need to respond to the high prevalence of STIs among AGYW in ASM. The risk factors for STIs identified in the study were adulthood; having primary education; engaging in risky behavior such alcohol consumption; and transactional. These factors constitute multiple and intersecting vulnerabilities that require both preventive and curative interventions targeting ASM AGYW in particular, ASM male workers and host communities in general. Awareness raising messages should emphasize behavioral change with respect to abstinence from alcohol misuse, abstinence from transactional sex by opting for viable alternative sources of income, safer sex, and prevention of sexual violence. The Ministry of Health and key development partners should partner with other relevant government and non-government stakeholders in designing the interventions which should not only target AGYW, but also ASM male workers. A multi sectoral approach that effectively engages key stakeholders, including mining host communities, is essential given the close interrelations between gender, health and economic aspects of the AGYW’s lives.

## Data Availability

All datasets for the study are available from the corresponding author on reasonable request.

## Abbreviations

AGYW: Adolescent girls and young women
ASM: Artisanal and small-scale mining
SRH: Sexual and reproductive health
STI: Sexually transmitted infection
